# Evaporative destabilization of a salt crust with branched pattern formation

**DOI:** 10.1038/s41598-023-31640-6

**Published:** 2023-03-29

**Authors:** G. Licsandru, C. Noiriel, P. Duru, S. Geoffroy, A. Abou-Chakra, M. Prat

**Affiliations:** 1 Institut de Mécanique Des Fluides de Toulouse (IMFT), Université de Toulouse, CNRS–Toulouse, Toulouse, France; 2 Géosciences Environnement Toulouse (GET), Observatoire Midi Pyrénées, Université Paul Sabatier, CNRS, IRD, CNES, Université de Toulouse, Toulouse, France; 3 LMDC (Laboratoire Matériaux Et Durabilité Des Constructions), Université de Toulouse, INSAT, UPS, Toulouse, France

**Keywords:** Engineering, Chemical engineering, Civil engineering

## Abstract

The impact of salt crust formation over porous media on water evaporation is an important issue in relation with the water cycle, agriculture, building sciences and more. The salt crust is not a simple accumulation of salt crystals at the porous medium surface but undergoes complex dynamics with possible air gap formation between the crust and the porous medium surface. We report on experiments that allow to identify various crust evolution regimes depending on the competition between evaporation and vapor condensation. The various regimes are summarized in a diagram. We focus on the regime where dissolution–precipitation processes lead to the upward displacement of the salt crust and the generation of a branched pattern. It is shown that the branched pattern results from the crust upper surface destabilization whereas the crust lower surface remains essentially flat. We show that the resulting branched efflorescence salt crust is heterogeneous with a greater porosity in the salt fingers. This leads to the preferential drying of the salt fingers followed by a period in which the crust morphology change only occurs in the salt crust lower region. The salt crust eventually tends toward a frozen state where no visible change occurs in the salt crust morphology, but without blocking the evaporation. These findings provide in-depth insights into the salt crust dynamics and pave the way for the better understanding of the impact of efflorescence salt crusts on evaporation and the development of predictive models.

## Introduction

Salt crusts have emerged as a specific and important research topic in the area of soil sciences^1–3^, environmental sciences^4,5^, building sciences^6^ and heritage conservation^7,8^. The salt crusts often form as a result of evaporation and in many cases, they represent a nuisance for buildings. Evaporation induces upward flow in the porous medium directed toward the evaporative surface. Dissolved salts are transported through the flow and tend to concentrate at the porous medium surface where evaporation takes place. When a sufficient ion concentration is reached, salt crystallization occurs and salt efflorescence develops. Associated important phenomena are the salt crust impact on evaporation and the ions transport in the underlying porous media as well as on the possible superficial degradation of the porous substrate. In this context, the salt crust morphology and its dynamic evolution are among the most important open questions since they reflect the whole complexity of the various processes at play. Sometimes, the crust is compact, almost flat^9,10^, but it can be also quite rough^11^ with occasionally bulges in some places^12^. In some cases, the crust is characterized by very-well defined patterns^13^, such as the polygonal cell pattern at the surface of some salt playas^14^. Under other circumstances salt domes form^3,15^. Since salt crusts can be submitted to relatively harsh conditions, such as high temperature variations and intense solar radiations, one could expect that the salt crust evolution at a soil surface or on a building material and the associated change in its morphology are essentially due to mechanical effects, such as underneath gas pressure generation or thermal expansion and shrinkage. Although these effects might play a role under field conditions, we report in this article on an experiment where the salt crust morphology change is quite important but not due to mechanical effects. The observed evolution in the crust morphology is analyzed and explained by coupled transport and dissolution (deliquescence)–precipitation processes. The experiment is performed under quasi-isothermal laboratory conditions at room temperature. It is expected that the processes involve in the observed morphology changes are also key processes in salt crust evolution in nature. In the reported experiment, the salt crust morphology changes from compact to branched.

**Figure 1:**
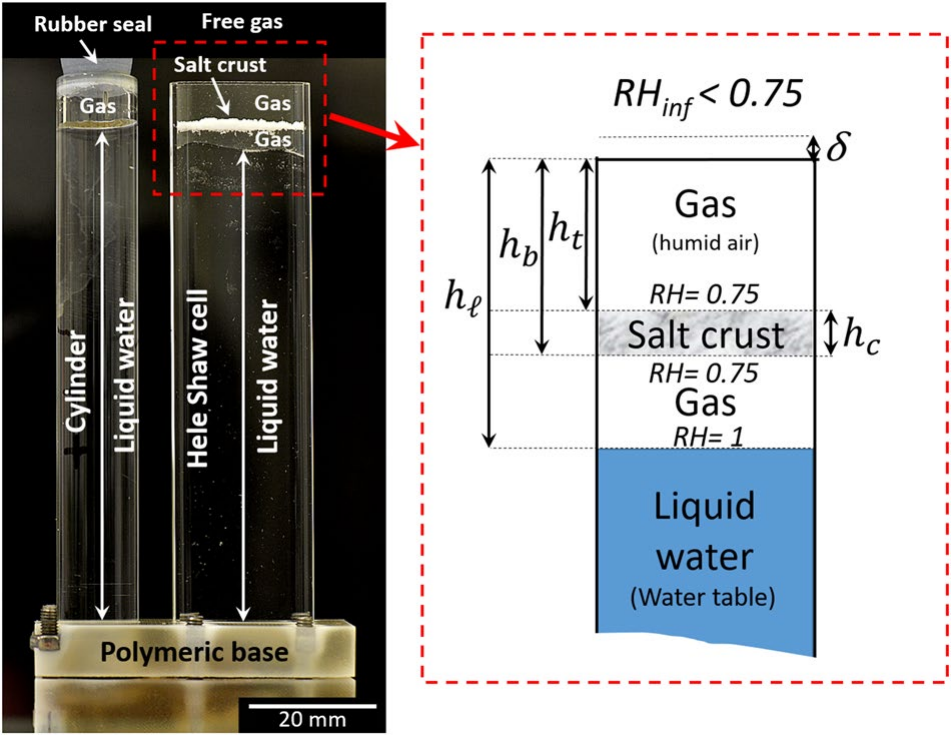
Experimental set-up. A salt crust is suspended in a Hele-Shaw cell (20 mm wide, 100 mm high, with an aperture of 2 mm) with liquid water at some distance below the crust. The cell is open at the top to a relatively dry air, which induces evaporation. The Hele-Shaw cell is connected through its polymeric base to a cylinder containing liquid water.

The experiment is performed in the same set-up as the one used in two previous studies^16,17^. As illustrated in Fig. 1 and described in more detail in the next section, the crust is set in a Hele-Shaw cell open at its top end and with a water table set at some distance below the crust bottom surface. Compared to the experiment reported and analyzed in^16^, the only difference lies in the initial positions of the crust and the water table within the cell. Whereas these positions in^16^ led to a regime in which no significant change in the crust morphology could be observed, the crust morphology change is tremendous in the present experiment. This sensitivity of the crust morphology to these parameters finally led to change them more significantly in two additional experiments briefly described in the last part of the present paper. Combined with the experiment reported in^16^, this led to identify various crust dynamics regimes, depending on the evaporation rate from the crust and the water absorption rate by the crust, which are summarized in a phase diagram presented also in the present paper. It can be also noted that the crust morphology and associated dynamic regime in^17^ was the same as in^16^, a regime referred to as stable. The important difference in^17^ lies in the observation and analysis of the detachment of the crust from the porous substrate over which the crust initially forms. In the experiments discussed here and in^16^, the focus is on the crust dynamics. Contrary to^17^, there is no porous substrate left in the cell in these experiments.

## Experiment

A 18 days experiment is performed, in which the evolution of a NaCl salt crust can be observed accurately and the factors controlling its dynamics can be assessed. As shown in Fig. 1, the NaCl salt crust is suspended in a Hele-Shaw cell (Fig. 1). The procedure to obtain a suspended crust is described in the section Materials and Methods at the end of the article. After the isolated salt crust in the Hele-Shaw cell is obtained, pure water is introduced in the Hele-Shaw cell via the adjacent cylindrical tube shown in Fig. 1 up to a desired distance below the crust and the latter is exposed to evaporation. In the experiment, the water level in the Hele-Shaw cell is set initially at a distance of 3 mm from the crust bottom surface. As can be seen from Fig. 1, the liquid gas interface in the cell is not flat. As explained in the section Materials and Methods, the cell is rendered hydrophobic by silanization. The fact that the interface is not flat is attributed to local variations in the contact angle. Also, the crust surfaces shown in Fig. 1 are not flat. As a result, the distances considered in the articles and indicated in Fig. 1 are average distances over 13 vertical lines uniformly distributed over the cell width (see “Materials and methods” section and Fig. 5a). The whole set-up is placed in an enclosure of controlled relative humidity (${RH}_{inf}\approx$ 39%) and temperature (*T*≈22 °C), which are recorded all along the experiment.

## Results

### Salt crust evolution

**Figure 2:**
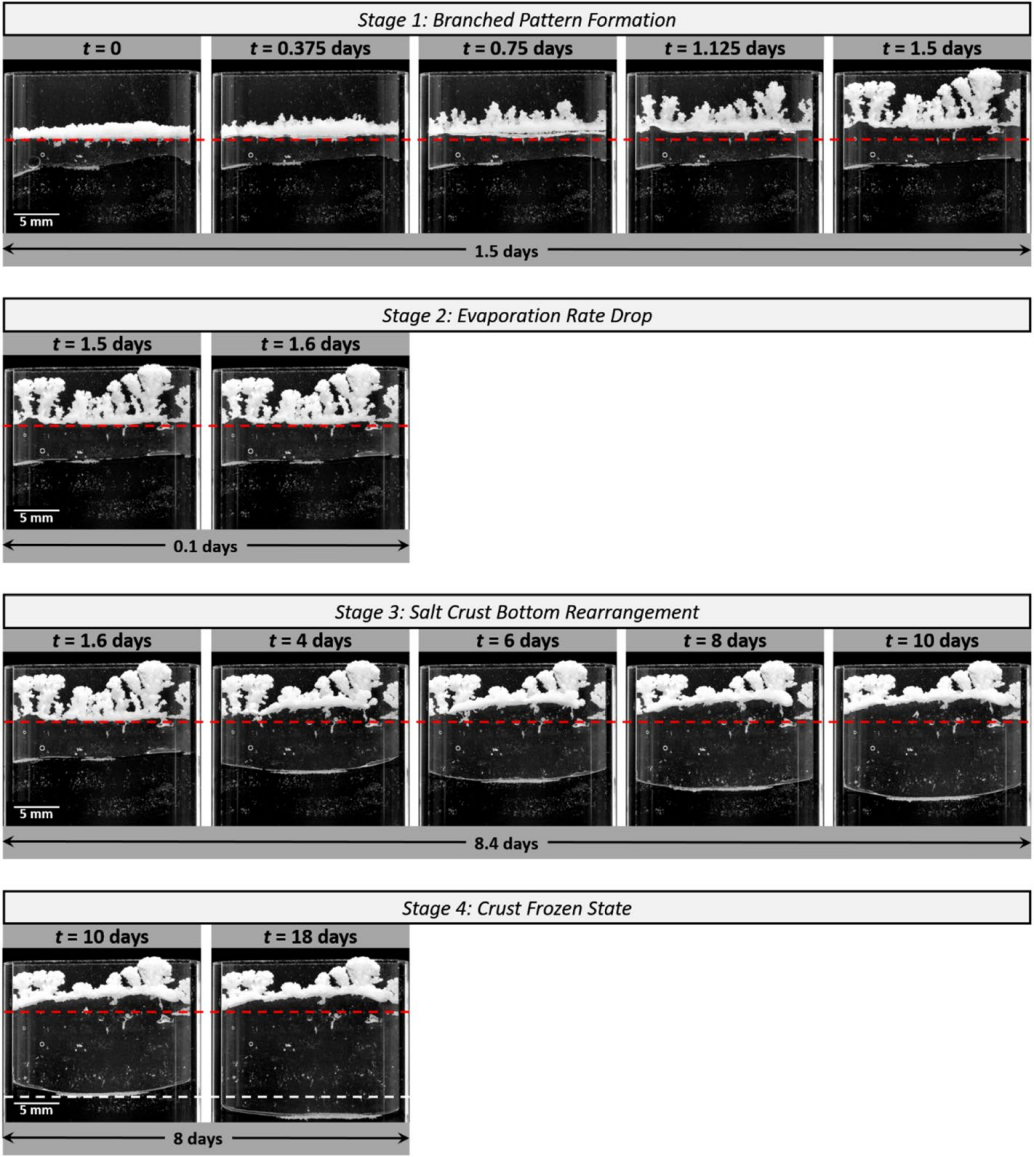
Pictures of the salt crust in the Hele-Shaw cell. Illustration of the various stages in the evolution of the crust identified from both the crust morphology modification and evaporation rate variation. The red horizontal dashed lines are guide to the eye to see better the crust evolution and its upward migration. The white objects on the liquid–gas interface or attached to the walls (more visible in the supplementary movies) correspond to residual glass beads.

The salt crust evolution is displayed in Fig. 2. As can be seen, the crust moves upward in the cell. The observed migration of the crust is consistent with previous works^16,17^, where the upward crust displacement was analyzed as a coupled process of transport phenomena and dissolution–precipitation mechanisms.

The upward migration results from relative humidity difference between the NaCl-saturated solution at the surface of the crust (*RH*≈0.75^18^) and the top of the water head in the cell (*RH* = 1). The relative humidity difference induces water vapor transport by diffusion between the pure liquid water surface in the cell and the crust bottom surface. Hence, the crust absorbs pure water from the reservoir as a result of water vapor condensation, causing dissolution of the crust bottom. Simultaneously, the crust top loses water by evaporation. Dissolved species at the crust bottom, i.e., Na^+^ and Cl^−^, are transported upward in crust pore network, until they reprecipitate at the crust top. As a result, new layers of crystals form at the crust top while dissolution progresses at the crust bottom. It is important to notice that this analysis and description of the salt crust upward migration is purely based on transport phenomena and dissolution—precipitation processes and do not involve mechanical considerations, i.e. stress generation. In particular, although the cell walls are hydrophobic (see “Materials and Methods” section), this description does not require the consideration of interactions between crystals and hydrophobic surfaces^19,20^ in order to be representative of most applications, which usually do not involve hydrophobic surfaces.

However, contrary to the experiments presented in^16,17^, where the salt crust remained compact, the crust morphology considerably changes in the present experiment. Thick salt fingers develop upward and a branched morphology is eventually obtained. In contrast, the salt crust bottom surface remains flat. Also, Fig. 2 shows that salt fingering eventually stops. More precisely, the branched pattern development occurs over a relatively short period of about 1.5 days at the beginning of the experiment (stage 1 in Fig. 2). Then, change in the crust morphology takes place in the crust bottom region over a period of about 8 days while no visible change in the salt crust top region can be noticed (stage 3 in Fig. 2). Then, during the last 8 days of the experiment, the liquid water in the cell continues to evaporate (as can be seen from the receding liquid water level position in the cell) whereas no change in the crust morphology is observed (stage 4 in Fig. 2). The branched pattern formation is a rapid process compared to the subsequent rearrangement of the crust bottom region and eventually, the crust reaches a frozen state.

### Crust migration

#### Absorption and evaporation kinetics

**Figure 3:**
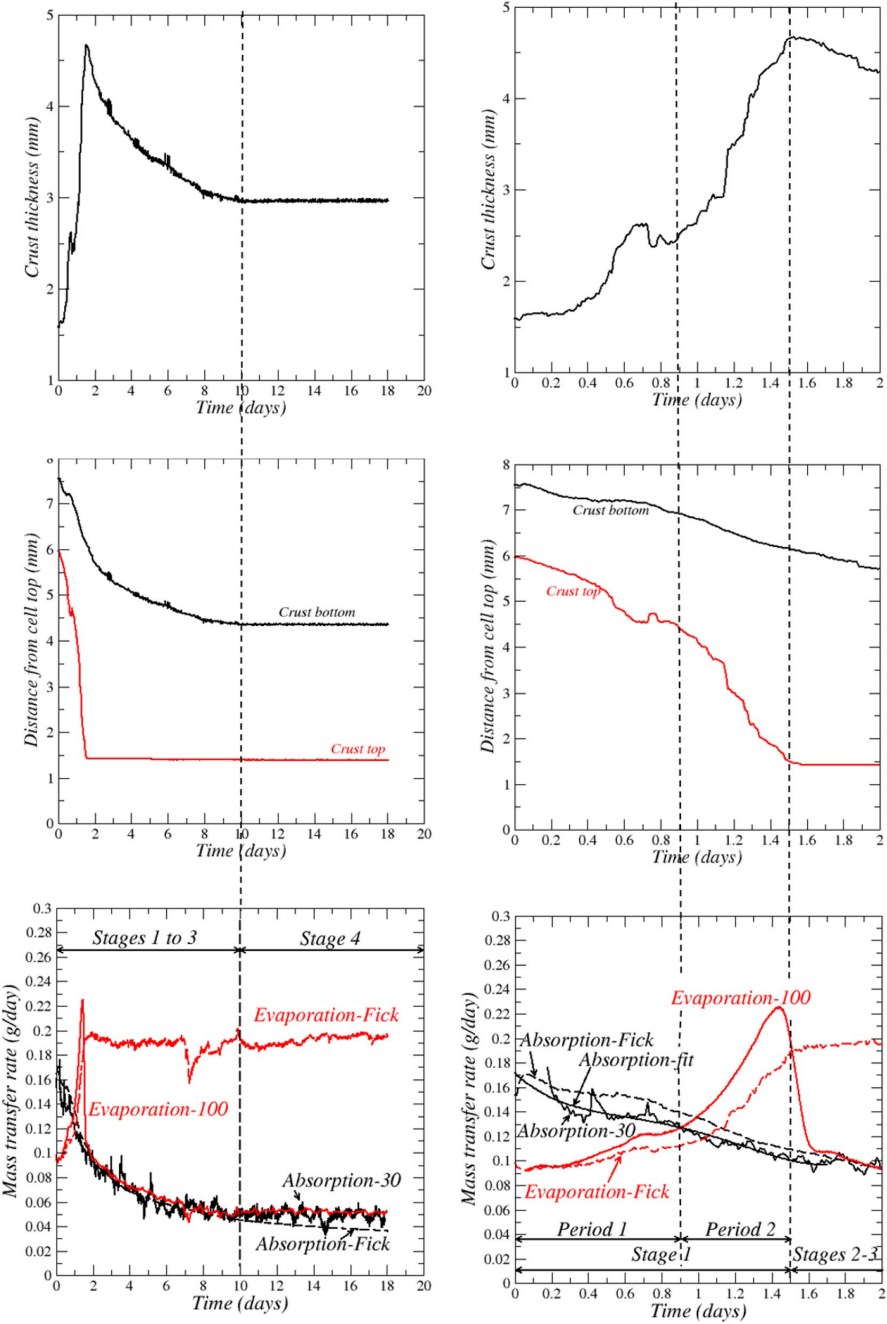
Evolution of salt crust thickness, crust top and bottom surfaces mean positions, potential evaporation (“Evaporation–Fick”), absorption and evaporation rates. The solid curves labelled “Absorption-30”, “Absorption-Fit” and “Evaporation-100” correspond to measurements (see the “Materials and Methods” section for details on how these curves are obtained). The dashed curves labelled “Evaporation-Fick” and “Absorption-Fick” are determined from Fick’s law (see text). The figures in the right column are a zoom over the experiment first two days.

Evolution of salt crust thickness, crust top and bottom surfaces mean positions, absorption and evaporation rates are shown in Fig. 3. The absorption rate is determined from the liquid level variations in the cell and adjacent cylinder (Fig. 1) while the evaporation rate is determined from the set-up weight variation. This gives the curves labelled “*Evaporation-100*”, “*Absorption-30”* and “*Absorption-fit*” in Fig. 3 (see the section “Materials and Methods” for details). The figures in the right column in Fig. 3 are a zoom over the experiment first two days. The crust thickness (Fig. 3) increases during stage 1 to a maximum reached at the end of stage 1 (day 1.5), then decreases during stages 1 and 3 and eventually reaches a constant value. The thickness increase in stage 1 results from the faster upward displacement of the crust top surface compared to the bottom surface. In contrast, the thickness decrease in the third stage results from the crust bottom upward displacement while the crust top remains immobile.

The absence of visible change in the salt crust corresponds in Fig. 3 to the stage after day 10 (stage 4) where there is no further displacement of both the salt crust bottom and top surfaces. As shown by the zoom in Fig. 3 (right column), two periods can be distinguished in stage 1. In the period 1 of stage 1 up to day 0.9, the absorption rate is greater than the evaporation rate. The second period in stage 1, from day 0.9 to day 1.5, is characterized by the strong increase in the evaporation rate, which becomes much greater than the absorption rate.

In the second and third stages from day 1.5 to day 10, the evaporation rate drops and become comparable to the absorption rate. The crust top surface does not move anymore in stage 3 whereas the crust bottom surface moves upward. The evaporation rate and the absorption rate are still comparable in the last stage (stage 4), from day 10 to day 18, but contrary to the third stage, the crust bottom surface is immobile and no change in the crust morphology is visible (Fig. 2).

## Analyses and explanations

### Crust thickness increase and absorption and evaporation

The initial stage (stage 1) of increasing salt crust thickness can be first discussed from simple mass conservation considerations. The salt mass in the crust is expressed as $m_{{salt}} \approx A\left( {\varepsilon \rho _{l} C_{{sat}} + \left( {1 - \varepsilon } \right)\rho _{{cr}} } \right)h_{c}$, where ${h}_{c}$, is the crust thickness (Fig. 1), ${\rho }_{l}$ is the solution density (${\rho }_{l}=\frac{{\rho }_{w}}{(1-0.7C)}$ where ${\rho }_{w}$ is the density of pure water), ${C}_{sat}$ is the dissolved sat mass fraction in the solution assumed everywhere closed to the solubility (${C}_{sat}$=0.264^21^), $\varepsilon$ is the crust porosity, ${\rho }_{cr}$ is the crystal (halite) density (${\rho }_{cr}$ = 2163 kg/m^3^^22^). Expressing that the salt mass is constant, i.e. $\frac{{dm}_{salt}}{dt}=0$, yields $\frac{1}{{h}_{c}}\frac{{dh}_{c}}{dt}=\frac{\left({\rho }_{cr}-{\rho }_{l}{C}_{sat}\right)}{\left(\varepsilon {\rho }_{l}{C}_{sat}+\left(1-\varepsilon \right){\rho }_{cr}\right)}\frac{d\varepsilon }{dt}$ . Similarly, the water mass in the crust is expressed as ${m}_{water}\approx A {{\rho }_{l}{(1-C}_{sat})\varepsilon h}_{c}$, which leads to $\frac{{dm}_{water}}{dt}=A {{\rho }_{l}{(1-C}_{sat})(\varepsilon \frac{{dh}_{c}}{dt}+ h}_{c}\frac{d\varepsilon }{dt})={J}_{abs.}-{J}_{evap.}$ , where ${J}_{abs.}$ is the water vapor condensation rate at the crust bottom surface and ${J}_{evap.}$ is the evaporation rate at the crust top surface. Combining these equations leads to $\frac{d{h}_{c}}{dt}=\frac{\left(1-\frac{{\rho }_{l}{C}_{sat}}{{\rho }_{cr}}\right)}{A{\rho }_{l}{(1-C}_{sat})}\left({J}_{abs.}-{J}_{evap.}\right)$. Accordingly, the salt crust thickness should increase when ${J}_{abs.}>{J}_{evap.}$ , i.e. when the salt crust gains water, and should decrease when ${J}_{abs.}<{J}_{evap.}$, i.e. when the salt crust loses water. Also, from the above equations, the crust porosity would increase when ${J}_{abs.}>{J}_{evap.}$ and would decrease when ${J}_{abs.}<{J}_{evap.}$

As can be seen, these considerations do not match with the results in Fig. 3 (right column). In the first period of stage 1 up to *t* ≈0.9 days, thickness increases and the absorption rate is indeed greater than the evaporation rate, i.e. ${J}_{abs}>{J}_{evap.}$. However, in the second period of stage 1 (between about *t*≈0.9 days and *t*≈1.5 days) the crust thickness continues increasing and the branched pattern continues developing whereas ${J}_{abs}<{J}_{evap.}$ Each period of stage 1 is now discussed in more detail.

#### **Stage 1 in excess absorption period (0**$\le$***t***$\le$**0.9 days).**

**Figure 4:**
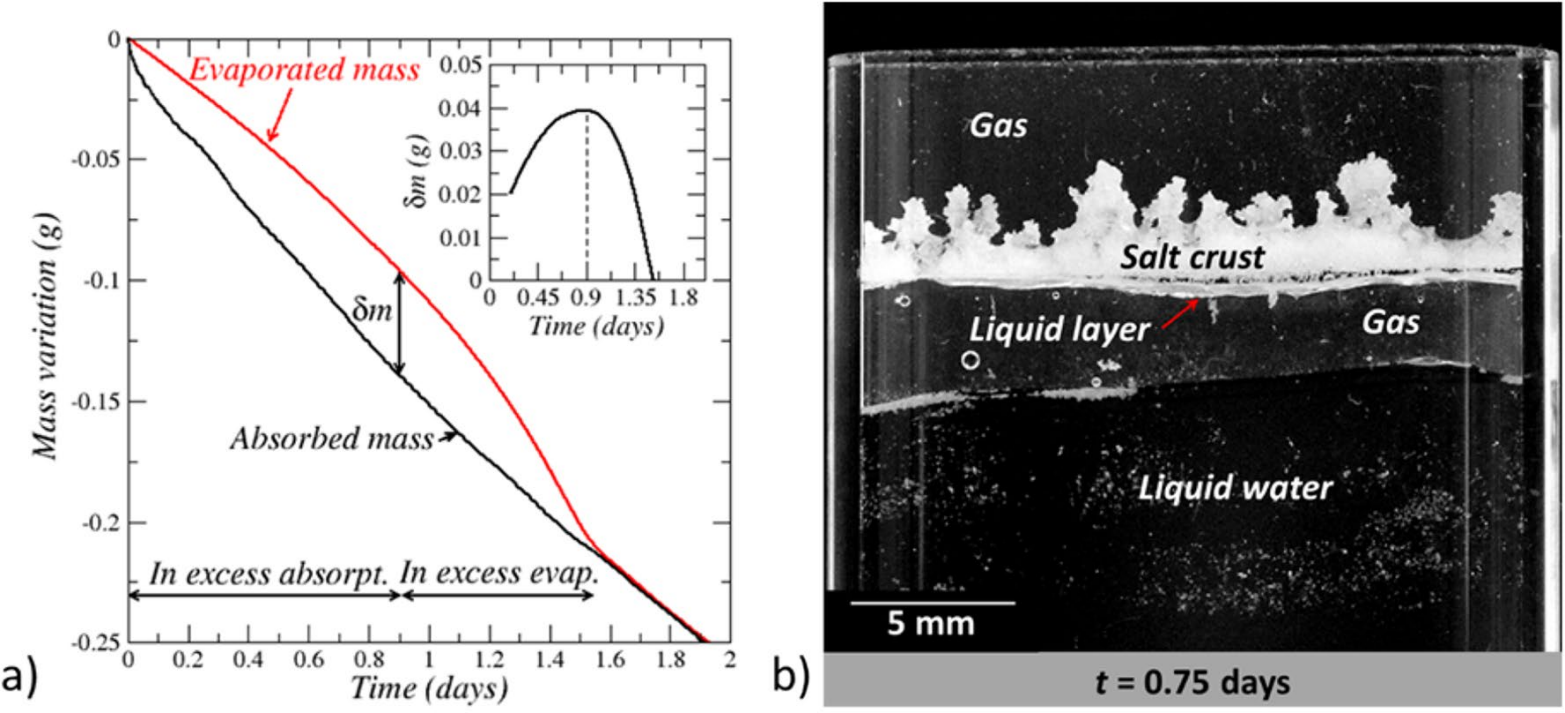
Absorbed and evaporated masses. (**a**) Variation of absorbed and evaporated masses over the first two days, (**b**) In excess absorption period: formation of a solution liquid layer underneath the salt crust.

 Figure 3 shows that absorption rate at the crust bottom is greater than the evaporation rate at the crust top from the start of the experiment to about *t* = 0.9 days. Consequently, the net mass of water inside the salt crust increases. Water absorption takes place at the salt crust bottom, but is limited by the evaporation rate. As a result, a layer of solution forms at the crust bottom. This liquid layer is visible in Fig. 4b. The liquid layer is not fully attached to the salt crust even if it remains hydraulically connected to it. From the absorbed and evaporated masses (Fig. 4a), the maximum water mass gained by the salt crust is determined, i.e., $\delta m$≈0.04 g (inset in Fig. 4a) corresponding to an average solution layer thickness of about 0.7 mm. Since this thickness is greater than the thickness of the liquid layer visible in Fig. 4b (about 0.44 mm), it can be concluded that the complementary fraction of water, i.e., about 40% of the absorbed water, contributes to the development of the branched pattern, i.e. to the expansion of the region occupied by the salt crust in the cell.

#### **Stage 1 in excess strong evaporation period (0.9 day **$\le$** t**$\le$**1.5 days).**

 The liquid layer underneath the crust forms a water reservoir in addition to the water present in the salt crust pore space. This reservoir gradually disappears as the liquid layer is absorbed into the salt crust as a result of the evaporation in excess. The results plotted in Fig. 4a consistently indicate that the strong evaporation period ends when all the liquid layer has been absorbed into the crust and the corresponding liquid water mass has evaporated. Prior to that, the strong increase in the evaporation rate indicates that the factor limiting the evaporation is the diffusive water vapor transport from the crust top. Water transport inside the crust is not limiting. This is supported by the evaporation rate computation from Fick’s law assuming that the vapor pressure over the crust top surface is the saturated water vapor pressure (*p*_*vs*_ $\approx$ 0.75 ${p}_{vsat}$ for a NaCl saturated solution where ${p}_{vsat}$ is the saturated vapor pressure for pure water). Hence, the evaporation rate is estimated from Fick’s law as ${J}_{evap-Fick}={A D}_{v}\frac{{M}_{v}}{RT}{p}_{vsat}\frac{\left(0.75 -{RH}_{inf}\right)}{{h}_{t}+\delta }$ by combining the following equations: ${J}_{evap-Fick}={A D}_{v}\frac{{M}_{v}}{RT}{p}_{vsat}\frac{\left(0.75 -{RH}_{cell-top}\right)}{{h}_{t}}$ and ${J}_{evap-Fick}={A D}_{v}\frac{{M}_{v}}{RT}{p}_{vsat}\frac{\left({RH}_{cell-top}-{RH}_{inf}\right)}{\delta }$ where *A* is the cell cross-section surface area (*A* = 0.004 m^2^), *D*_*v*_ is the water vapor molecular diffusion coefficient ($\approx$ 2.7 × 10^−5^ m^2^/s ^23^,), *R* is the universal gas constant, *M*_*v*_ is the vapor molecular weight,${h}_{t}$ is the mean distance between the crust top surface and cell top (Fig. 1), ${RH}_{cell-top}$ is the relative humidity at the cell top, ${RH}_{inf}$ is the relative humidity in the surrounding air. The first equation describes the water vapor diffusive transport between the crust top surface and the cell top whereas the second equation describes the mass transfer between the cell top and the surrounding air. The latter is obviously three-dimensional. A simplified approach consists in introducing a mass transfer coefficient at the cell top. Here, the mass transfer coefficient is expressed as the equivalent thickness $\delta$ of an additional gas layer of cross-section area $A$. From comparison with the evaporation rate computed from the set-up mass variations at the experiment very beginning, it was found that $\delta$≈3 mm. This value is consistent with the value for tubes^24^ indicating that $\delta$ is on the order of the tube aperture (the cell aperture is 2 mm). The evaporation rate so determined from Fick’s law is also referred to as the potential evaporation by analogy with the potential evaporation in soil physics^25^. Here, the potential evaporation is the evaporation assuming that the salt crust top surface is covered by a liquid film of NaCl saturated solution. This leads to the result depicted in Fig. 3 (bottom panel in right column) where it can be seen that the evaporation rate computed from Fick’s law follows the same trend as the evaporation rate determined from the weight variation measurement until the evaporation rate is maximum. The strong increase in the evaporation rate results from the upward migration of the crust. The lower the distance between, the salt crust top and the cell top, the higher the evaporation rate. The evaporation rate determination from Fick’s law using the mean distance between the salt crust top surface and the cell top is of course only an approximation since the pattern is highly branched. This explains the evaporation rate underestimation from Fick’s law observed over the strong evaporation rate period. This does not call into question the interpretation: the transport inside the crust is not limiting and the vapor pressure over the crust top surface is the saturated vapor pressure for a NaCl saturated solution, i.e. ${p}_{vs}=$ 0.75 ${p}_{vsat}$, over the considered period of stage 1.

### Branched pattern formation

**Figure 5:**
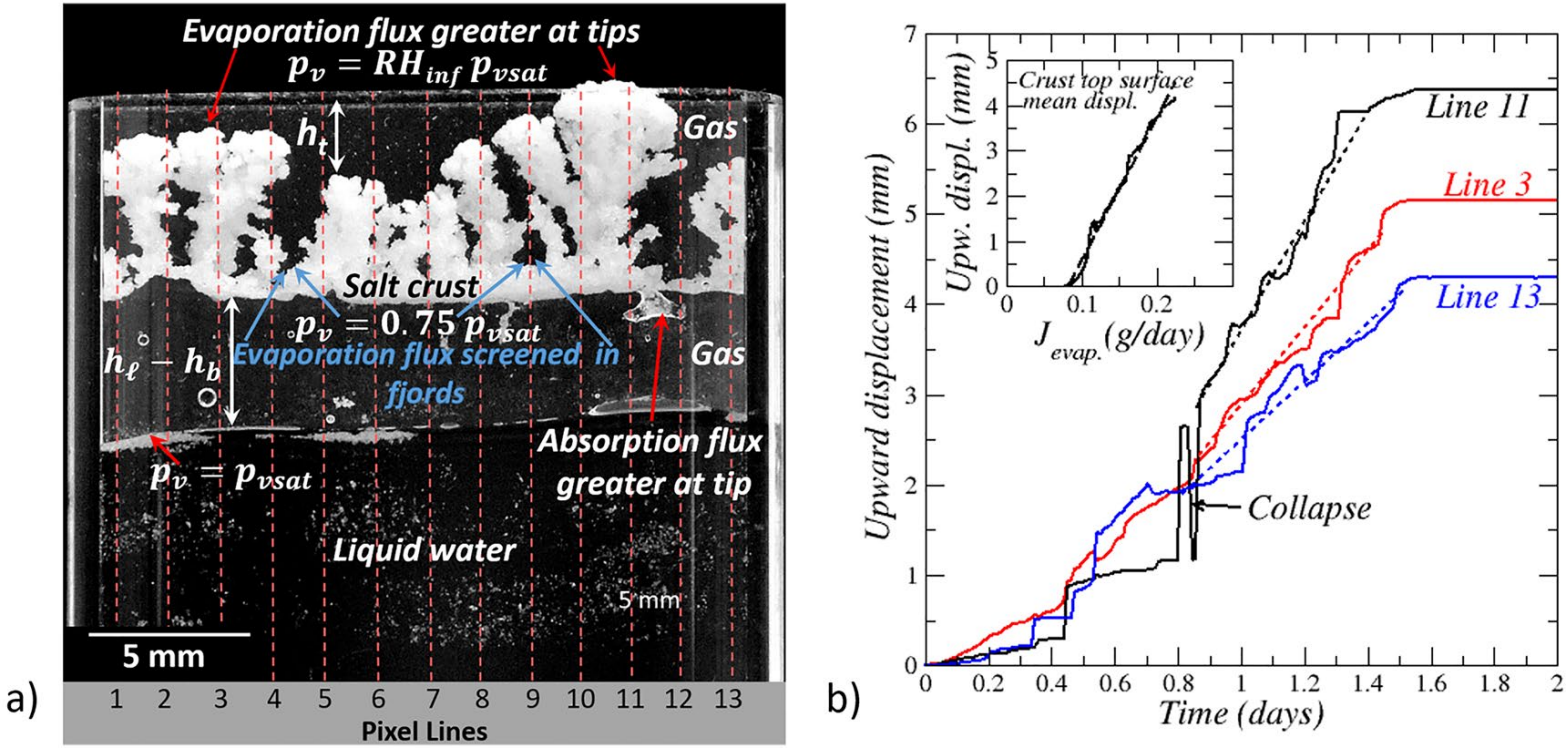
Evaporation flux distribution in stage 2. (**a**) Schematic of evaporation flux distribution at crust top surface and absorption flux distribution at crust bottom surface during the salt crust destabilization period; vertical pixel lines considered in the image processing (see the “Materials and Methods “section). It can be noted that the distance ${h}_{t}$ can be locally equal to zero since some fingers reach the cell top. The average distance over the vertical lines shown in the figure is however greater than zero, (**b**) salt crust top surface displacement along lines 3, 11 and 13 (selected because corresponding to finger tips). The inset shows the crust top surface mean displacement as a function of the evaporation rate (up to day 1.5).

The most spectacular feature in the experiment is the development of the branched salt pattern during stage 1. The destabilization of the flat crust is explained as follows. The crust top surface local growth rate can be expressed using the relationship derived in^16^, namely $\frac{d{{\varvec{X}}}_{t}}{dt}$ = $\frac{{C}_{sat}}{{\rho }_{cr}\left(1-\varepsilon \right)\left(1-{C}_{sat}\right)}\left[\frac{Da}{1+Da}\right]{{\varvec{j}}}_{evap.}.\mathbf{n}$ where ${{\varvec{j}}}_{evap.}$ is the local evaporation flux, ${{\varvec{X}}}_{t}$ is a position vector at the surface and $\mathbf{n}$ is a unit normal vector at the crust top surface, $Da$ is the Damköhler number characterizing the competition between the precipitation reaction and the diffusive ion transport^16^. Thus $\frac{d{{\varvec{X}}}_{t}}{dt} \propto {{\varvec{j}}}_{evap.}$ with ${{\varvec{j}}}_{evap.}\propto {1/h}_{t}$ (the closer the considered point at the surface to the cell top, the greater is the local evaporation flux according to Fick’s law). Since the evaporation flux is greater at the crust top surface most advanced points, defined as the points the closest to the cell top, these points move faster than the points of the surface further away from the top cell. This effect is enhanced by the screening effect in the fjords which further reduces the evaporation flux in the fjords^26^ (as indicated in Fig. 5a, the fjords refer to the regions of the crust top surface least advanced points).

The screening effect refers to the fact that the evaporation flux is much lower in the fjords. In other words, the destabilization is due to the dependence of the crust top surface local displacement speed with the local evaporation flux as schematically illustrated in Fig. 5a and the fact that the closer the surface to the cell top, the greater is the evaporation flux. The upward displacement of a few markers at the salt crust top surface corresponding to salt finger tips is shown in Fig. 5b. The tip displacement speed (dashed line slopes in Fig. 5b) is consistent with the scaling $\frac{d{{\varvec{X}}}_{t}}{dt}$ $\propto {{\varvec{j}}}_{evap.}$ since the higher the salt fingertip position in the cell, the higher is the evaporation flux and the higher is the average displacement speed in Fig. 5b. As indicated by Fig. 5b, a finger can occasionally collapse during its growth (visible also in the experiment video in Supplementary). After the collapse, it resumes growing. Nevertheless, the dominant feature is the expansion of the region occupied by the salt crust in the cell.

The inset in Fig. 5b further illustrates the linear dependence of the salt crust top surface mean displacement with the evaporation rate consistently with the expected scaling $\frac{d{{\varvec{X}}}_{t}}{dt}$ $\propto {{\varvec{j}}}_{evap.}$.

The branched pattern development continues as long as evaporation is not limited. When all the water in excess at the crust bottom is consumed, the branched pattern development stops since, as indicated in Fig. 3, the absorption rate is significantly lower than the evaporation rate when the latter reaches its maximum. Hence, evaporation cannot be balanced by absorption once the water in excess at the bottom has been totally absorbed into the crust.

Similar considerations explain why the crust bottom surface displacement is stable. Using again the expression derived in^16^, the displacement of a point located at the crust bottom surface is expressed as $\frac{d{{\varvec{X}}}_{b}}{dt}$ = $\frac{{C}_{sat}}{{\rho }_{cr}\left(1-\varepsilon \right)\left(1-{C}_{sat}\right)}\left[\frac{Da}{1+Da}\right]{{\varvec{j}}}_{abs.}.\mathbf{n}$**.** Thus, $\frac{d{{\varvec{X}}}_{b}}{dt} \propto {{\varvec{j}}}_{abs.}$ with ${{\varvec{j}}}_{abs.}\propto 1/{(h}_{l}-{h}_{b})$ (the smaller the distance between the liquid level in the cell and the crust bottom surface, the greater is the absorption flux). As a result, the absorption flux is greater at the least advanced points of the crust bottom surface, defined as the closest points to the cell bottom. This is schematically indicated in Fig. 5a. The dissolution is therefore more effective at these points, which contributes to smooth out the bottom surface.

### Branched pattern textural contrast

**Figure 6:**
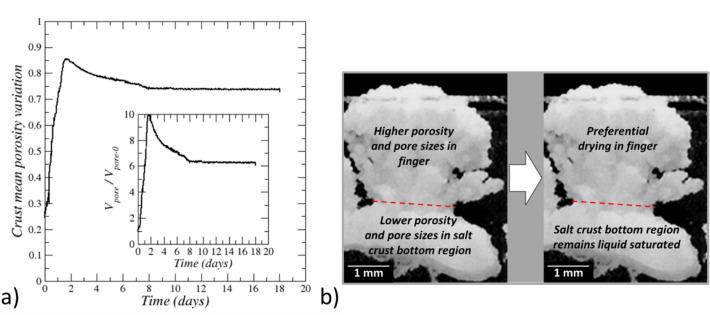
Crust porosity variation. (**a**) Salt crust average porosity variation (assuming ε = 0.25 at *t* = 0). The inset shows the ratio between the corresponding salt crust pore volume and the salt crust pore volume at *t* = 0. (**b**) Schematic of porosity distribution in the branched salt crust and preferential drying in fingers due to textural contrast between the salt fingers and the salt crust bottom region.

As a result of the branched pattern formation, the crust spreads over a large region compared to the initial state (compared the crust at *t* = 0 and *t* = 1.5 days in Fig. 2). The ratio of the crust surface areas between the two pictures, i.e. at *t* = 1.5 days and *t* = 0, is 2.9. Since the net salt mass does not change, this implies a significant increase of the crust porosity over stage 1. From salt mass conservation, the crust porosity can be expressed as $\varepsilon =\frac{{(\varepsilon }_{0}\left({\rho }_{l}{C}_{sat}-{\rho }_{cr}\right)+{\rho }_{cr})\left(\frac{{V}_{c0}}{{V}_{c}}\right)-{\rho }_{cr}}{\left({\rho }_{l}{C}_{sat}-{\rho }_{cr}\right)}$ where ${\varepsilon }_{0}$ is the salt crust initial porosity, ${V}_{c}$ is the salt crust volume and ${V}_{c0}$ is the salt crust volume at *t* = 0. Assuming that $\frac{{V}_{c0}}{{V}_{c}}\approx \frac{{A}_{c0}}{{A}_{c}}$, where ${A}_{c}$ is the salt crust area in the pictures and ${A}_{c0}$ is the salt crust area in the picture at *t* = 0 and taking ${\varepsilon }_{0}$=0.25 as an example, one obtains the salt crust mean porosity variation displayed in Fig. 6a from the determination of ${A}_{c}$ by image processing. The mean porosity variation is significant, with a maximum increase in the porosity by a factor 3.4.

Consequently, the pore space volume has increased by a factor 10 when the salt crust volume has reached its maximum value (inset in Fig. 6a). However, examination of the experiment video (Supplementary) suggests that the fingers do not always fill entirely the cell gap. Hence, the approximation $\frac{{V}_{c0}}{{V}_{c}}\approx \frac{{A}_{c0}}{{A}_{c}}$ tends to underestimate the volume ratio $\frac{{V}_{c0}}{{V}_{c}}$. Thus, the increase in the porosity and the pore volume is a bit overestimated in Fig. 6. Nevertheless, this does not change the conclusion: the increase in the porosity and the pore volume is quite significant. Since the overall salt crust porosity increases during the branched pattern development and the crust new regions correspond to the salt fingers, it can be inferred that the porosity is higher in the salt fingers that in the crust bottom region. Although a greater porosity does not necessarily imply greater pore sizes, we surmise that here the higher porosity is associated with larger pore sizes. In other words, the branched salt crust is a heterogeneous porous crust characterized by a textural contrast between the salt finger region, where the porosity and the pores are larger, and the region underneath where the porosity and the pores are smaller. This textural contrast is sketched in Fig. 6b. The evidence of the textural contrast is supported by the fact that change in the crust during stage 3 (see Fig. 2), only occurs in the bottom region of the crust whereas no change are visible in the top part, indicating that liquid is present in the bottom region but not in the top region.

#### Stage 2: Evaporation rate drop. Drying of salt fingers (1.5 days $\le$***t***$\le$ 1.6 days)

At the very beginning of this stage (stage 2) the evaporation rate at the crust top is much greater than the absorption rate at the crust bottom. Then the evaporation rate drops significantly in less than 3 h (Fig. 3) and becomes rapidly comparable to the absorption rate (Fig. 3). As shown in Fig. 2, there is no noticeable change in the salt crust morphology over this short period. As depicted in Fig. 4a, the maximum water mass *δm* gained by the crust during the branched pattern development has evaporated at the end of the considered stage. Thus, the water mass net gain is zero over this stage. Furthermore, since the evaporation and absorption rates are very close at the end of stage 2, i.e. at *t*≈1.6 days (Fig. 3 bottom panel on the left column), the conclusion is therefore that the mass of liquid in the crust at the end of the considered stage is about equal to the liquid water mass at the experiment beginning. Since the pore space volume has significantly increased (Fig. 6a) whereas the liquid mass is about the same as in the initial crust, this means that the branched crust is not fully saturated at the end of stage 2 and that the average saturation is significantly less than the liquid saturation in the initial crust (the latter being considered as fully saturated). In other words, the salt crust has dried out. It is known from previous studies on drying of porous media with textural contrast^10,27,28^, that the gas preferentially invades the region of larger pore sizes in the drying process, thus here the salt fingers. This is a consequence of the lower invasion capillary pressure threshold in larger pores (according to Young–Laplace law, the invasion capillary pressure threshold of a pore is inversely proportional to its size)^26^. This situation is sketched in Fig. 6b. Hence stage 2 between about *t* = 1.5 days and *t* = 1.6 days is characterized by the preferential drying of the salt fingers. Also, as a result of the salt finger desaturation, the crust top surface stops moving whereas the crust bottom surface continues to moves upward (Fig. 3).

#### Stage 3. Crust bottom region rearrangement period (1.6 days $\le$***t***$\le$ 10 days)

**Figure 7:**
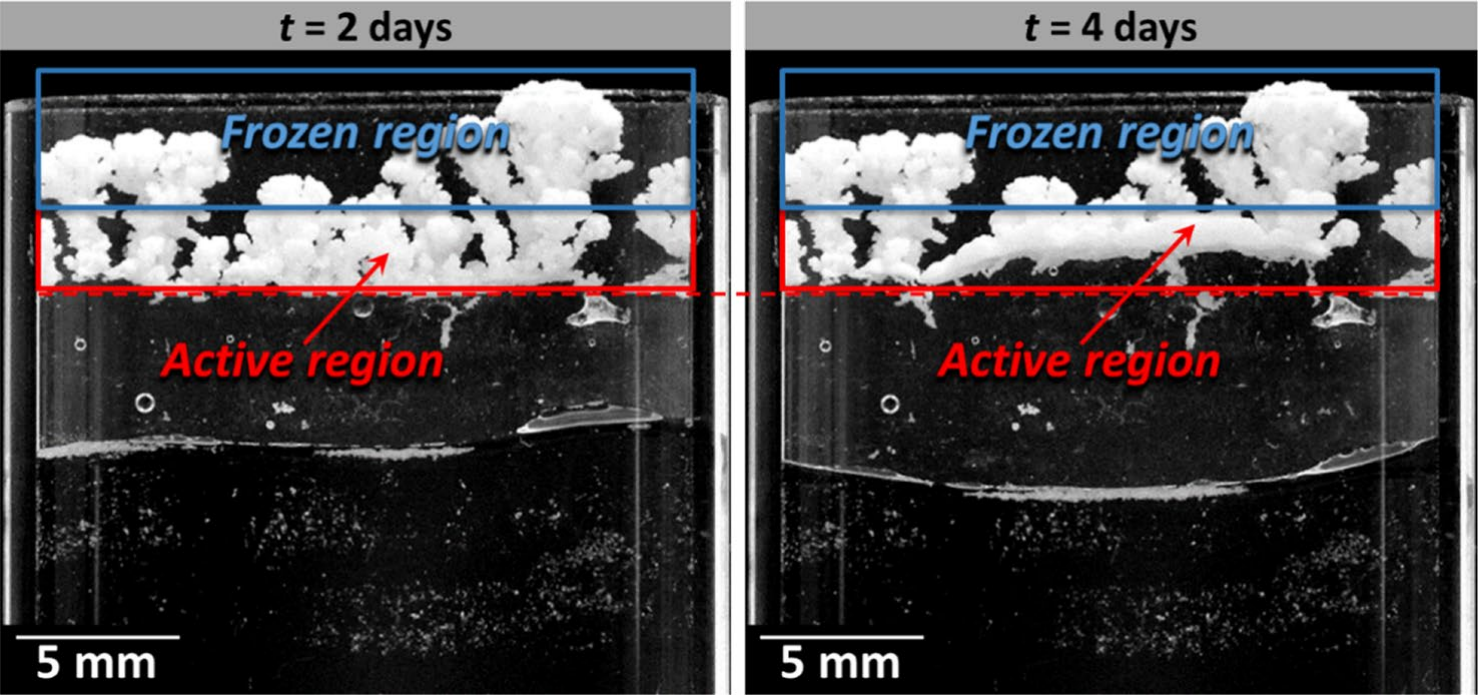
Salt crust active and frozen regions. Changes in the crust morphology only occur in the active region between the two times indicated in the pictures (stage 3).

Examining the images after the evaporation rate drop leads to identify two distinct regions consistently with the preferential desaturation of the salt fingers: (i) the immobile region and (ii) the active region (Fig. 7).

**Figure 8:**
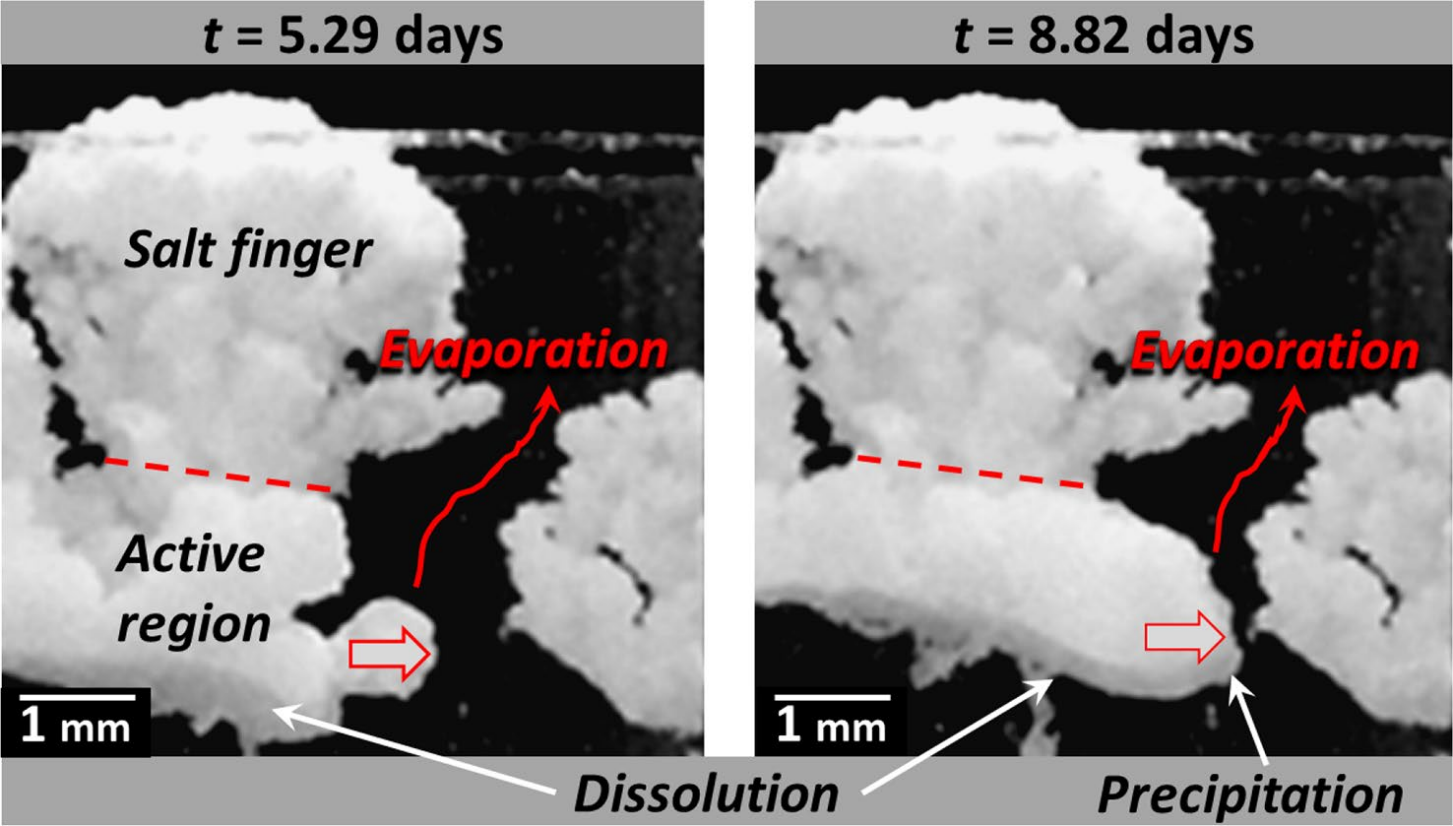
Lateral growth in the salt crust bottom region indicating evaporation and a high liquid saturation in this region of the crust (stage 3).

No change is visible in the salt finger region, i.e. the salt crust upper region, which is therefore referred to as the immobile or “frozen” region. The salt crust morphology change occurs in the lower region, referred to as the active region in Fig. 7. Since no further salt crust displacement occurs in the upper region, evaporation takes place deeper in the crust where displacements are visible, i.e. on the top and sides of the active region. This is further illustrated in Fig. 8 showing a situation of crust lateral growth in the active region.

As can be seen from Fig. 3 (left column bottom panel), applying Fick’s law assuming that the water vapor partial pressure is *p*_*vs*_ = 0.75 *p*_*vsat*_ over the salt crust bottom surface, i.e. computing ${J}_{abs}$ as ${J}_{abs-Fick}=A{D}_{v}\frac{{M}_{v}}{RT}{p}_{vsat}\frac{\left(1-0.75\right)}{{h}_{l}-{h}_{b}}$ , where ${h}_{l}-{h}_{b}$ is the distance between the crust bottom surface and the liquid surface in the cell bottom (Figs. 1 and 5), leads to a fair estimate of the absorption rate during stage 3. Thus, the vapor partial pressure is close to the saturated partial pressure for a NaCl saturated solution, i.e. 0.75 *p*_*vsat*_, over the salt crust bottom surface, consistently with a liquid saturated salt crust active region.

#### Stage 4: salt crust “frozen” state (10 days $${\varvec{t}}{\varvec{o}}$$$$\le$$***t***$$\le$$ 18 days)

**Figure 9:**
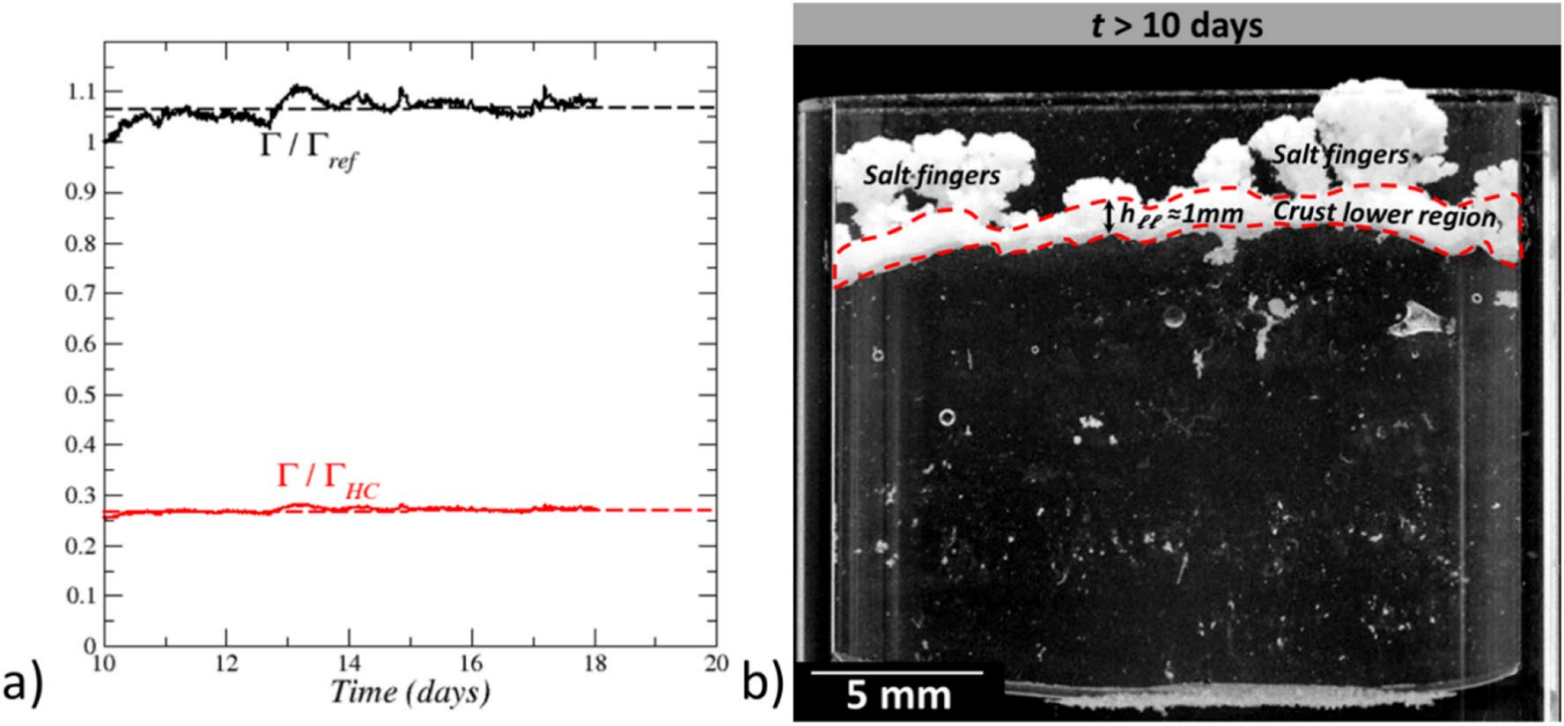
Salt crust diffusive conductance. (**a**) Salt crust diffusive conductance as a function of time in stage 4; ${\Gamma }_{ref}$ is the conductance at *t* = 10 days; ${\Gamma }_{HC}$ is the conductance in the Hele-Shaw cell free of crust; (**b**) Salt crust for *t* > 10 days with tentative separation between the salt finger region (crust upper region) and the salt crust lower region (region within the red dashed frame).

This stage is characterized by the salt crust full frozen state. No change in the crust morphology is visible but evaporation still occurs (Fig. 3). It can be observed from Fig. 3 that the absorption rate computed from the Fick’s law as ${J}_{abs-Fick}=A{D}_{v}\frac{{M}_{v}}{RT}{p}_{vsat}\frac{\left(1-0.75\right)}{{h}_{l}-{h}_{b}}$ underestimates the absorption rate. This is an indication that the relative humidity at the crust bottom surface ${RH}_{crust-bottom}$ is less than 0.75, i.e. the value for a NaCl saturated solution. Since the bottom surface was a dissolution zone in the preceding stage, it is unlikely that the pores are sufficiently small for the Kelvin effect, i.e. the change in vapor pressure due to a curved liquid–vapor interface, to explain the decrease in the relative humidity at the crust bottom surface (the curvature radius must be less than about 50 nm for the Kelvin effect to have a significant impact^29^ whereas flat interfaces are rather expected in dissolution region) . Thus, the most likely scenario is that the water saturation is quite low in the crust, most probably close to zero (dry crust). Assuming that the crust is dry, the mass transfer rate between the crust bottom surface and the cell top can be expressed as $J\approx {A \varepsilon D}_{eff}\frac{{M}_{v}}{RT}{p}_{vsat}\frac{\left({ RH}_{crust-bottom}-{RH}_{inf}\right)}{{h}_{b}}$ where ${h}_{b}$ is the distance between the crust bottom surface and the cell top (Fig. 1), $\Gamma =\frac{{ \varepsilon D}_{eff}}{{h}_{b}}$, can be interpreted as the water vapor diffusive conductance of the zone occupied by the salt crust in the Hele-Shaw cell. From Fick’s law, $J$ is also equal to $J=A{D}_{v}\frac{{M}_{v}}{RT}{p}_{vsat}\frac{\left(1-{ RH}_{crust-bottom}\right)}{{h}_{l}-{h}_{b}}$ . Combining the latter two equations leads to express $\Gamma$ as $\Gamma =\frac{{ \varepsilon D}_{eff}}{{h}_{b}}={\left[\frac{A{M}_{v}{p}_{vsat}\left(1-{RH}_{inf}\right)}{JRT}-\frac{{h}_{l}-{h}_b}{{D}_{v}}\right]}^{-1}$, which can be computed from the experimental data. If the crust is dry, the diffusive conductance $\Gamma$ must not vary as the liquid water level recedes into the cell. The variation of $\Gamma$ so obtained (using a linear fit of the variation of *J* over the considered period) is shown in Fig. 9a. As can be seen, $\Gamma$ increases slightly over the first day of the considered period and then is reasonably constant. The interpretation is that the crust dries out at the beginning of stage 4 in about 1 day. Then no significant change in the liquid content occurs. The latter is expected to be very low so that the diffusive transport in vapor phase through the crust is the dominant water transport mechanism. In Fig. 9, $\Gamma$ is compared to the diffusive conductance in the Hele-Shaw cell in the absence of crust, i.e. ${\Gamma }_{HC}=\frac{{ D}_{v}}{{h}_{b}}$ . As can be seen, $\Gamma / {\Gamma }_{HC}\approx 0.27$. Although the salt crust occupies only a fraction of the upper region of the cell, this relatively high value is an indication that the crust resistance to vapor diffusion is not very high compared to diffusion in the free gas. This was expected as regards the salt fingers since the salt finger porosity is expected to be high as discussed before.

To get further insights into the crust porosity, suppose that the crust diffusive resistance is essentially due to the lower layer resulting from the dissolution–precipitation process occurring in the active region in the previous stage (stage 3). Then, if the salt finger resistance is neglected, the diffusive conductance of the lower layer (sketched in Fig. 9b) can be estimated as $\frac{{\varepsilon D}_{eff-ll}}{{h}_{ll}}={\left[\frac{A{M}_{v}{p}_{vsat}\left(1-{RH}_{inf}\right)}{JRT}-\frac{{h}_{l}-{h}_{b}}{{D}_{v}}-\frac{{(h}_{b}-{h}_{ll})}{{D}_{v}}\right]}^{-1}$ using as before an approach of resistances in series and where ${h}_{ll}$ is the mean thickness of the crust lower layer (Fig. 9b). For ${h}_{ll}\approx 1$ mm (Fig. 9b), this leads to $\frac{{\varepsilon D}_{eff-ll}}{{D}_{v}}\approx 0.008$. Using the classical relationship $\frac{{\varepsilon D}_{eff-ll}}{{D}_{v}}={\varepsilon }^{1.33}$^30^, this gives $\varepsilon \approx 0.15$. This represents a lower bound estimate of the crust lower layer porosity since we have neglected here the diffusive resistance due to the salt fingers. Combined with Fig. 7a, this further illustrates the textural contrast between the crust lower layer and the salt fingers.

Finally, it can be noticed that the diffusive resistance of the salt crust estimated here is much less than the one reported in^31^ for instance. This is an indication that the salt crust properties can significantly vary depending on the salt crust formation condition and its dynamic evolution.

## Discussion and conclusion

We have presented an experiment of upward displacement of a (NaCl) salt crust in Hele-Shaw cell during which a branched pattern develops. Branched patterns are observed in many areas of physics, material science, biology and the earth sciences and have been the subject of many studies, e.g.^32–37^. However, as pointed out in^38^, branched patterns usually form toward the material source. Typical examples are the dendrite growth in solidification^39^ or the diffusion limited aggregation process^33^. Here, the situation is different because the coupling between evaporation, capillarity and ion transport within the growing porous salt structure leads to growth in the direction opposite to the material source, the salt in our experiment. Branched NaCl salt structures were reported in^9^ but only over coarse porous media and as well individualized salt branches referenced to as “patchy” efflorescence because this type of efflorescence does not cover entirely the porous medium surface and thus does not form a crust. Since salt crusts preferentially develops over fine porous media^9^, the present study shows that branched structures can be expected not only at the surface of coarse media but also at the surface of fine porous media as the result of the salt crust destabilization.

The branched pattern reported here is significantly different from the compact crusts observed in previous experiments^16,17^ using the same experimental set-up. All these experiments put forward the key role played by the relative contribution of water absorption and evaporation rates in the salt crust dynamics. The present experiment is characterized by an initial period where the absorption rate is higher than the evaporation rate, resulting in the accumulation of liquid water in excess at the crust bottom. The condensation layer at the crust bottom is a key factor for the destabilization of the crust during its upward displacement and the branched pattern generation. The liquid layer together with the branched pattern development induce a higher evaporation rate than the water vapor condensation rate (absorption). Once the condensation layer has been entirely absorbed into the crust as the result of evaporation in excess, the salt crust tends toward a quasi-equilibrium state where no further evolution of the crust morphology is noticed. This quasi-equilibrium state is reached via a relatively complex sequence of preferential drying of the salt fingers and rearrangement of the salt crust bottom part.

Since the crust morphology evolution is markedly different in the present experiment compared to the evolution reported in^16^ or^17^, it is interesting to investigate further the various regimes that can be expected from our experimental set-up. It can be first recalled that the evaporation and absorption rates are comparable over a long period and the crust moves upward without significant morphology change in^16,17^. This displacement regime can thus be considered as stable. In the present experiment, the absorption rate is sufficiently larger than the evaporation rate over a sufficiently long initial period for the branching pattern to occur. This clearly indicates that the ratio between the evaporation rate from the crust and the water absorption rate by the crust, i.e. the net balance between evaporation and absorption, is a key parameter.

The initial value of this dimensionless ratio estimated from Fick’s law at the beginning of the experiments is reported in Table 1. Note that the crust is assumed fully saturated by a saturated NaCl aqueous solution (which corresponds therefore to ${RH}_{crust}=0.75$ at the crust top and bottom surface) to compute this ratio. The ratio $\frac{{J}_{evap}}{{J}_{abs.}}$ is thus estimated as $\frac{{J}_{evap}}{{J}_{abs.}}\approx \frac{({RH}_{crust}-{RH}_{inf})}{(1-{RH}_{crust})}\frac{({h}_{l}-{h}_{b})}{({h}_{t}+\delta )}$. When this ratio is sufficiently close to 1, the stable regime is obtained. When this ratio is lower ($\approx$ 0.5 in the case of the branched pattern experiment discussed in the paper) the branched pattern is obtained. However, when this ratio is sufficiently low, the evaporation rate is expected to be not sufficient to compensate the absorption in excess and the crust dissolution can be expected.

**Table 1:** Initial values of the relative humidity in the enclosure, distance ${h}_{l}-{h}_{b}$ between the crust bottom surface and water table (Fig. 1), distance ${h}_{t}$ between the crust top surface and the cell top (Fig. 1), and evaporation rate-absorption rate ratio estimated from Fick’s laws assuming the crust pores occupied by a saturated NaCl aqueous solution for the four experiments considered in the discussion.

	${RH}_{inf}$	${h}_{l}-{h}_{b}$(mm)	${h}_{t}$(mm)	${J}_{evap}/{J}_{abs.}$
Full dissolution regime	0.4	2.33	15.6	0.18
Branched regime	0.38	3	6	0.5
Stable regime^16^	0.38	9	16.9	0.67
Stagnant regime	0.39	47	16.9	3.4

**Figure 10:**
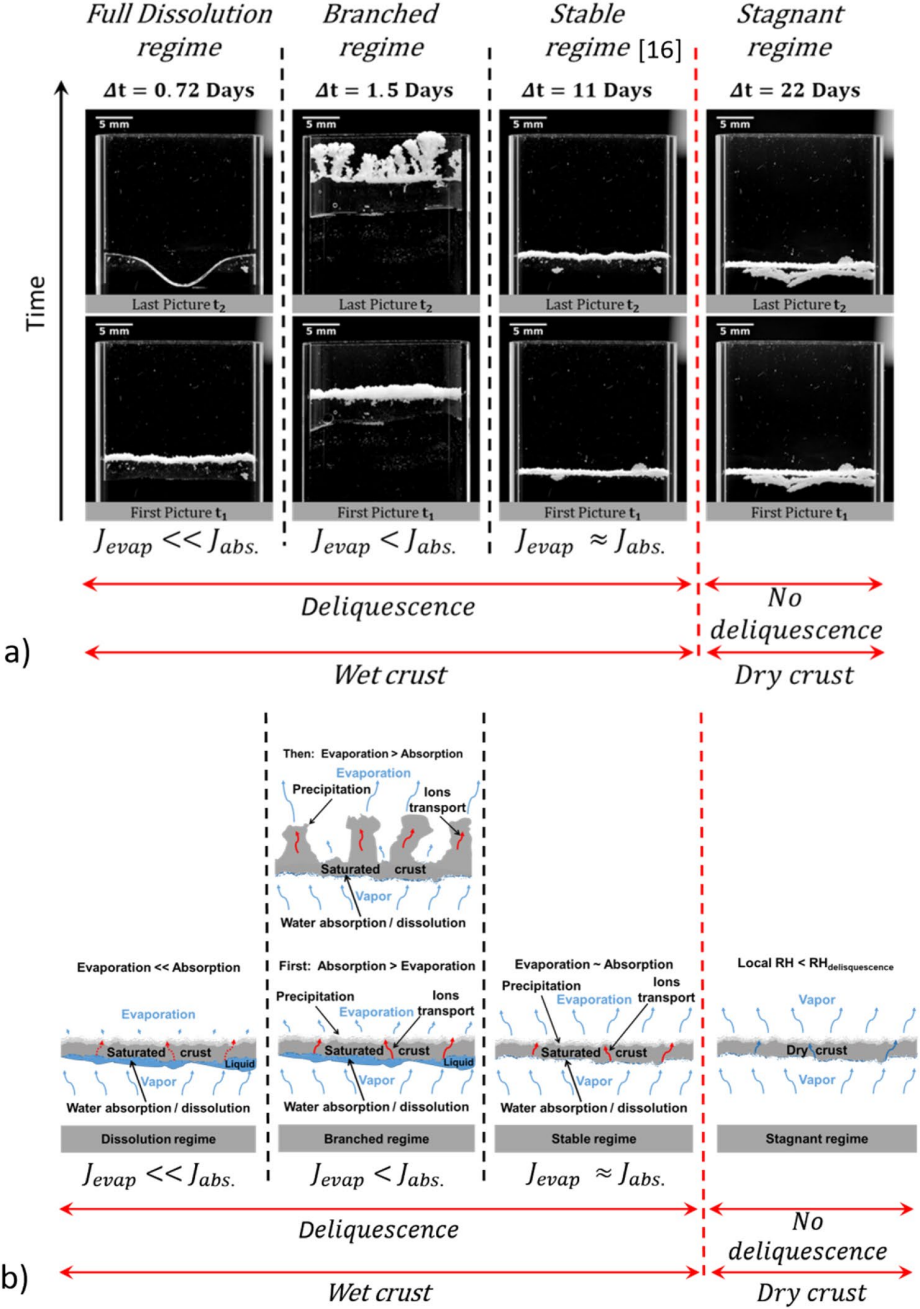
Salt crust dynamics phase diagram. (**a**) Each column of two pictures correspond to a separate experiment, i.e. given initial distances between the crust top surface and the cell top, i.e.${h}_{t}$, and the crust bottom surface and the water level (water table), i.e. ${h}_{l}-{h}_{b}$. For a given column, the picture on the bottom corresponds to the initial image of the crust in the cell whereas the image on the top is the image after the indicated elapsed time $\Delta t$, (**b**) Schematic of the different regimes with indication of main transport and physicochemical mechanisms. “Local RH” means the relative humidity in the vicinity of the crust.

This is illustrated with an additional experiment where $\frac{{J}_{evap}}{{J}_{abs.}} \approx 0.18.$ As indicated in Table 1, this ratio was obtained by setting the crust deeper in the cell than for the branched pattern experiment so as to reduce the evaporation rate and with the position of the water table a bit closer to the crust than for the branched pattern experiment. Under these circumstances, the crust full dissolution occurs (see movie in Supplementary). This is the full dissolution regime. By contrast, when the liquid level in the cell is too low, the potential evaporation, i.e. the evaporation computed assuming the crust saturated by a saturated aqueous NaCl solution, is much higher than the potential absorption and no evolution of the crust occurs. The crust stays immobile. This corresponds to the stagnant regime. The various regimes as schematically summarized in Fig. 10b with an indication of main transport and physicochemical mechanisms controlling the crust dynamics.

The existence of the stagnant regime illustrates the fact that the various regimes first depend on whether the conditions are such that crystal deliquescence can occur. A crystal of soluble salt deliquesces when it is exposed to a vapor pressure equal or greater than the vapor pressure of its saturated solution at the existing temperature^40^ ($\approx$ 0.75 ${p}_{vsat}$ for NaCl^18^). When the liquid level is sufficiently far below the crust and the relative humidity at the cell top is low, the relative humidity in the vicinity of the crust is less than 0.75 and no deliquescence occurs. The crust remains dry (neglecting the small amount of water that can be fixed by adsorption). This is the stagnant regime (Fig. 10). When the liquid level is sufficiently close to the crust and the relative humidity at the cell top sufficiently high, then deliquescence occurs and liquid water invades the crust pores. The next step, i.e. the crust evolution regime, depends on the relative contribution of the evaporation rate and the absorption rate as schematically indicated in Fig. 10 and Table 1 but also on the time period during which the absorption rate is higher than the evaporation rate. Nevertheless, the phase diagram shown in Fig. 10 is essentially qualitative. Additional experiments will be desirable to delineate in more detail the various dynamic regimes. Also, the various regimes were obtained by modifying the position of the crust in the cell and the position of the water table, i.e. the parameters ${h}_{l}-{h}_{b}$ and ${h}_{t}$ (Fig. 1). It would be interesting to extent and confirm the results presented here by playing also with the external relative humidity, i.e.${RH}_{inf}$, which is also a parameter affecting the ratio $\frac{{J}_{evap}}{{J}_{abs.}}$.

In summary, the present study contributes to elucidate the dynamics of salt crusts and of their observed morphology development. The model situation studied in the experiment can be encountered in nature in relation with the position of the water table in a soil for instance. Starting with an efflorescence salt crust already in place, the rise of the water table would play a role similar to the bottom liquid layer in the Hele-Shaw cell experiment. Also, together with the experiments analyzed in^16,17^, these findings suggest a simple and non-destructive method to control efflorescence salt crusts by adequate injection of pure water so as to induce the salt crust self-detachment from the material and maximize the crust porosity. This can be of interest for instance when the efflorescence salt formation is used as a low-cost method for recovering metals from wastes, such as mine tailings for instance^41^.

## Materials and methods

### How to obtain a suspended crust in the Hele-Shaw cell

 The cell is rendered hydrophobic by silanization so as to avoid salt creeping on the cell inner walls^42^ during the experiment. The procedure to obtain a suspended crust follows the procedure described in^16^. The Hele-Shaw is filled with glass beads (1–50 µm in diameter) up to certain level, saturated with a NaCl aqueous solution (of salt mass fraction equal to 25%) and submitted to evaporation. As a result of evaporation, a salt crust forms within the Hele-Shaw cell on top of the beads. After a certain time, the beads are carefully removed from the Hele-Shaw cell and a suspended layer is obtained. As detailed in^17^, this layer is actually a composite system formed by the assembly of two porous layers. The top layer essentially consists of NaCl crystals whereas the bottom layer is a mixture of glass beads and NaCl crystals. Then pure water is introduced at the bottom of the cell and the crust is exposed again to evaporation. As analysed in^17^, introduction of a water head below the crust causes the upward migration of salt crust up to the detachment of an essentially crystalline layer from the remaining glass bead layer. Then the remaining glass beads are removed from the Hele-Shaw cell, which therefore now only contains a salt crust, i.e. a porous layer whose matrix is essentially formed by halite crystals (a few small beads can, however, be trapped in this layer).

### Experiment reproducibility

In the present paper, three experiments have been presented; the one leading to the crust full dissolution, the one corresponding to the stagnant regime and the one leading to the branched pattern, which was analysed in much more detail. First, it can be noted that the branched pattern was observed in a separate pre-test experiment, which was a strong motivation for performing the more instrumented experiment analysed in the present paper. Also, the stable regime regime has been observed in two separate experiments^16,17^, in the experiment reported in^43^, as well as in several pre-test experiments. Furthermore, the various regimes summarized in the phase diagram also illustrates the overall consistency of all the experiments performed.

### Relative humidity and temperature control

#### Cell weight measurement

The Hele-Shaw cell is set in the balance proprietary enclosure (a Mettler-Toledo AX205 precision scale with readability up to 10^−5^ g was used) adapted in order to transform it into an environmental cell. This consists in inserting two medium petri dishes, filled with a saturated ${K}_{2}C{O}_{3} \mathrm{solution }(\approx RH=44 \%$^18^), that are suspended in the enclosure, in order to control the relative humidity, and the insertion of a humidity and temperature sensor (Rotronic Hygroclip SP05 probe). The temperature in the room is kept constant at about *T* ≈ 22 °C.

### Data acquisition

Several parameters are recorded during the experiment: (i) the mass of the set-up is measured every 100 s with the precision scale, (ii) the relative humidity in the enclosure and the temperature are measured every 1000 s and (iii) photographs of the set-up are taken every 1000 s in portrait mode with a Nikon D800E camera at a resolution of 7360 × 4912 pixels set outside of the enclosure.

Dedicated image processing techniques are used to determine distances in the cell and the adjacent cylinder with the aid of ImageJ Fiji software. One can refer to^17^ for details on the used image processing techniques. Distances in the cell, mean distance between the crust top (bottom respectively) surface and the cell top for instance, position of the water table in the cell, are averaged distances over the 13 vertical lines of pixels defined in Fig. 5a. A central vertical line of pixel is considered for determining the liquid level position in the cylinder. The salt crust mean thickness is the difference between the mean position of the salt crust top and bottom surfaces.

The evaporation rate at the crust top is computed from the set-up weight variation whereas the water absorption rate at the crust bottom is computed from the liquid level variations in the cell and the adjacent cylinder (Fig. 1). The evaporation rate is determined from the measured mass loss *m(t)* by a simple finite difference as ${J}_{evap}=-(m\left(t\right)-m\left(t-\Delta t\right))/\Delta t$, where $\Delta t$ is the elapsed time between two weight measurement. The results are then smoothed out by running averages. The running average is performed over intervals of 100 successive values. This is why the curve so obtained is labelled *“Evaporation–100”* in Fig. 3. The absorption rate is computed from the variations of the liquid levels in the cell and the adjacent cylinder as ${J}_{absorp.}=-\frac{d{m}_{level}}{dt}=-{\rho }_{l}\left(A\frac{{dh}_{lc}}{dt}+{A}_{cyl}\frac{{dh}_{lcyl.}}{dt}\right)$, where ${m}_{level}$ is the mass of liquid water at the bottom of the set-up (cell and cylinder). The latter is expressed as ${{m}_{level}= \rho }_{l} {(Ah}_{llcell}+{A}_{cyl}{h}_{llcyl.})$ where ${h}_{lc}$ and ${h}_{lcyl.}$ are the liquid heights in the Hele-Shaw cell and the feeding cylinder (visible in Fig. 1), respectively. These heights are determined as a function of time from the processed photographs; $A$ and ${A}_{cyl}$ are the Hele-Shaw cell and cylinder cross-section surface areas, respectively. The derivatives $\frac{{dh}_{lc}}{dt}$ and $\frac{{dh}_{lcyl.}}{dt}$ are computed by finite difference as $\frac{{dh}_{lc}}{dt}=\frac{{h}_{lc}\left(t+\Delta t\right)-{h}_{lc}(t)}{\Delta t}$ (and similar expression for $\frac{{dh}_{lcyl.}}{dt}$), where $\Delta t$ is here the elapsed time between two successive images. Then the absorption rate so obtained is smoothed out by running averages using intervals of 30 successive values. It is recalled that the mass is measured every 100 s while the levels are determined every 1000 s. This explains in part the difference in the length of the running average intervals between evaporation (100) and absorption (30). These interval lengths were determined after some trials when the obtained results were judged as sufficiently smoothed out. This yields the curve labelled “*Absorption-30*” in Fig. 3. For the zoom over stage 1 (Fig. 3, bottom panel on the right column), the absorption rate was also determined by deriving a polynomial fit of the curve ${m}_{level}(t)$ to further clarify the trend resulting from the running average procedure. This corresponds to the curve “*Absorption-fit*” in Fig. 3.

## Supplementary Information

Supplementary Information 1.

Supplementary Video 1.

Supplementary Video 2.

## Data Availability

All data needed to evaluate the conclusions in the paper are present in the paper and/or the Supplementary Materials. Data are available from the corresponding author on reasonable request.
