# Supplementary material for: Evaporative destabilization of a salt crust with branched pattern formation
Source: Sci Rep. 2023 Mar 29;13:5132. doi: 10.1038/s41598-023-31640-6 (PMC10060431; doi:10.1038/s41598-023-31640-6)
Supplement: Supplementary file 1 — Supplementary Information 1. [file 41598_2023_31640_MOESM1_ESM.docx]

**Supplementary information**

S1. Movie: Upward migration of salt crust due to dissolution-precipitation with branched pattern formation.

S2. Movie: Salt crust full dissolution regime.
